# Supplementary material for: Multi‐dataset identification of innovative feature genes and molecular mechanisms in keratoconus
Source: J Cell Mol Med. 2024 Sep 19;28(18):e70079. doi: 10.1111/jcmm.70079 (PMC11412914; doi:10.1111/jcmm.70079)
Supplement: Supplementary file 2 — Material S2. [file JCMM-28-e70079-s002.zip › 7.GSEA analysis/2.ATOH7/KEGG_HOMOLOGOUS_RECOMBINATION.html]

Details for gene set KEGG\_HOMOLOGOUS\_RECOMBINATION[GSEA]

|  || Dataset | ATOH7.group.cls#h\_versus\_l |
| Phenotype | group.cls#h\_versus\_l |
| Upregulated in class | l |
| GeneSet | KEGG\_HOMOLOGOUS\_RECOMBINATION |
| Enrichment Score (ES) | -0.30314153 |
| Normalized Enrichment Score (NES) | -1.0173495 |
| Nominal p-value | 0.43333334 |
| FDR q-value | 1.0 |
| FWER p-Value | 1.0 |
Table: GSEA Results Summary

  

Fig 1: Enrichment plot: KEGG\_HOMOLOGOUS\_RECOMBINATION      
 Profile of the Running ES Score & Positions of GeneSet Members on the Rank Ordered List

  

| SYMBOL | TITLE | RANK IN GENE LIST | RANK METRIC SCORE | RUNNING ES | CORE ENRICHMENT || 1 | RAD51 | na | 3872 | 0.241 | -0.0004 | Yes |
| 2 | RAD51D | na | 5714 | 0.179 | 0.0290 | Yes |
| 3 | NBN | na | 7019 | 0.143 | 0.0573 | Yes |
| 4 | EME1 | na | 7649 | 0.127 | 0.0976 | Yes |
| 5 | RAD54L | na | 9358 | 0.093 | 0.0911 | Yes |
| 6 | RPA3 | na | 10418 | 0.075 | 0.0950 | Yes |
| 7 | POLD3 | na | 11763 | 0.056 | 0.0820 | Yes |
| 8 | RAD51B | na | 12112 | 0.051 | 0.0955 | Yes |
| 9 | RAD52 | na | 12156 | 0.051 | 0.1175 | Yes |
| 10 | POLD2 | na | 12245 | 0.050 | 0.1378 | Yes |
| 11 | RPA2 | na | 12501 | 0.046 | 0.1517 | Yes |
| 12 | RPA1 | na | 13459 | 0.036 | 0.1406 | No |
| 13 | MUS81 | na | 14616 | 0.024 | 0.1186 | No |
| 14 | RAD51C | na | 16087 | 0.012 | 0.0819 | No |
| 15 | BRCA2 | na | 16341 | 0.010 | 0.0792 | No |
| 16 | RAD50 | na | 16590 | 0.008 | 0.0758 | No |
| 17 | BLM | na | 17597 | -0.001 | 0.0470 | No |
| 18 | TOP3B | na | 18907 | -0.012 | 0.0149 | No |
| 19 | TOP3A | na | 18922 | -0.012 | 0.0201 | No |
| 20 | POLD4 | na | 19537 | -0.017 | 0.0105 | No |
| 21 | RAD54B | na | 19854 | -0.020 | 0.0107 | No |
| 22 | POLD1 | na | 20660 | -0.028 | 0.0006 | No |
| 23 | SSBP1 | na | 20697 | -0.029 | 0.0127 | No |
| 24 | XRCC3 | na | 23414 | -0.061 | -0.0376 | No |
| 25 | XRCC2 | na | 32630 | -0.336 | -0.1481 | No |
| 26 | RPA4 | na | 33771 | -0.452 | 0.0276 | No |
Table: GSEA details [plain text format]

  

Fig 2: KEGG\_HOMOLOGOUS\_RECOMBINATION      
 Blue-Pink O' Gram in the Space of the Analyzed GeneSet

  

Fig 3: KEGG\_HOMOLOGOUS\_RECOMBINATION: Random ES distribution      
 Gene set null distribution of ES for **KEGG\_HOMOLOGOUS\_RECOMBINATION**
